# Book Reviews

**Published:** 1902-12

**Authors:** 


### BOOK REVIEWS.

A Text-book of Practical Therapeutics, with special reference to the application of remedial measures to disease, and their employment upon a rational bases. By Hobart Amory Hare, M.D., B.Sc., Professor of Therapeutics and Materia Medica in the Jefferson Medical College of Philadelphia; Physician to the Jefferson Medical College Hospital; One-time Clinical Professor of Diseases of Children in the University of Pennsylvania; Laureate of the Royal Academy of Medicine in Belgium, of the Medical Society of London; Corresponding Fellow of the Sociedad Espanola de Higiene of Madrid; author of a "Text Book on Practical Diagnosis," etc. *Ninth edition.* Enlarged, thoroughly revised and largely rewritten. Illustrated with 105 engravings and 4 colored plates. Published by Lea Brothers & Co., Philadelphia and New York, 1902.

The call for nine editions of this well-known work in ten years has afforded the author frequent opportunities for revision of the text, and incorporating all the new remedies and methods which have stood the test of clinical experience. Nearly 100 illustrations have been added, a large number of which show the actual application of the procedure described. In many respects the book can be considered almost a new one and is upon a subject of great interest and importance to all practitioners of medicine.

---

The Principles and Practice of Gynecology. For Students and Practitioners. By E. C. Dudley, A.M., M.D., Professor Gynecology, Northwestern University Medical School; Gynecologist to St. Luke and Wesley Hospitals, Chicago; Fellow of the American Gynecological Association; Corresponding Member of the Congrés Périodique International de Gynecologie et d'Obstetrique; ex-President of the Chicago Gynecological Society. *Third edition.* Revised and enlarged, with 474 illustrations, of which 60 are in colors and 22 full-page plates in colors and monochrome. Published by Lea Brothers & Co., Philadelphia and New York, 1902.

This is undoubtedly the most practicable treatise on gynecology in English. A thorough revision, including all the recent advances in this branch, has been made. Many chapters have been entirely rewritten, others rearranged or condensed, and about one hundred new pages have been added. Many of the illustrations used in previous editions have been replaced with new ones, showing the different procedures, consecutively, of the principal minor and major operations. The book is to be commended in every way and should be on the shelves of every general practitioner as well as the gynecologist.

---

PRACTICAL DIAGNOSIS: THE USE OF SYMPTOMS AND PHYSICAL SIGNS IN THE DIAGNOSIS OF DISEASE. *Fifth edition.* Revised and enlarged. By Hobart Amory Hare, M.D., B.Sc., Professor of Therapeutics in the Jefferson Medical College of Philadelphia; Physician to the Jefferson Medical College Hospital; one time Clinical Professor of the Diseases of Children in the University of Pennsylvania; Laureate of the Medical Society of London, of the Royal Academy of Medicine in Belgium; Corresponding Fellow of the Sociedad Espanol de la Higiene of Madrid; Member of the Association of American Physicians; Author of a Text-book of Practical Therapeutics. Illustrated with 236 engravings and 25 plates. Published by Lea Brothers & Co., Philadelphia and New York, 1902.

A book that has passed through five editions in six years certainly possesses merit of a marked degree, and furnishes a more correct conception of its true value, than the statement of any reviewer. The scope of the present edition has been broadened, and a large number of illustrations added, with a view of making the book as complete as possible. In its amplified form this edition should be quickly exhausted.

---

A TEXT-BOOK OF HISTOLOGY AND MICROSCOPIC ANATOMY OF THE HUMAN BODY, INCLUDING MICROSCOPIC TECHNIQUE. By Ladislaus Szymonowicz, M.D., A.O., Professor of Histology and Embryology in the University of Lemberg. Translated and edited by John Bruce MacCallum, M.D., Johns Hopkins University, Baltimore. Illustrated with 277 engravings, including 57 plates in colors and monochrome. Published by Lea Brothers & Co., Philadelphia and New York, 1902.

Anything coming from the pen of this well-known original investigator at once commands attention, and the appearance of this thoroughly modern text-book, in English, will no doubt promptly meet with the full degree of success to which it is so richly entitled. The illustrations are decidedly the best that we have seen in any work on this subject, and the entire book is gotten up in unusually handsome form. We heartily commend the work to the teacher, practitioner and student of American medicine.

PRACTICAL OBSTETRICS. A Text-book for Practitioners and Students. By Edward Reynolds, M.D., Visiting Surgeon to the Free Hospital for Women; Fellow of the American Gynecological Society; of the Obstetrical Society of Boston, etc.; formerly Instructor in Obstetrics in Harvard University, and Senior Physician to Out-Patients of the Boston Lying-In Hospital, and Franklin S. Newell, M.D., Assistant in Obstetrics and Gynecology in Harvard University; Physician to Out-Patients of the Boston Lying-In Hospital; Assistant Visiting Physician to the Boston City Hospital, in the Department of the Diseases of Women; Fellow of the Obstetrical Society of Boston, etc. Illustrated with 252 engravings and three colored plates. Published by Lea Brothers & Co., Philadelphia and New York, 1902.

The favorable reception accorded an earlier volume, aiming at rendering the technical details of obstetrical practice more accessible to the student, has encouraged the authors to prepare a more extended work on the same lines. Being founded upon an extended experience in clinical teaching, the general principles of the subject are rendered more intelligible and interesting than usual. The illustrations are profuse and appropriate.

---

PHYSICAL DIAGNOSIS. DISEASES OF THE THORACIC AND ABDOMINAL ORGANS. A Manual for Students and Physicians. By Egbert Le Fevre, M.D., Professor of Clinical Medicine and Associate Professor of Therapeutics in the University and Bellevue Hospital Medical College; Attending Physician to Bellevue and St. Luke's Hospitals; Consulting Physician to Beth-Israel Hospital; Member of the New York Academy of Medicine, etc. Illustrated with 74 engravings and 12 monochrome plates. Published by Lea Brothers & Co., Philadelphia and New York, 1902.

This excellent work has been prepared at the urgent request of a number of the former students of the distinguished author, who desired to have the methods used by him in teaching in permanent form. Especial emphasis has been laid on the altered anatomy of the organs under examination, and its relations to the physical signs. The respiratory and cardiac sounds, their production and their modifications, both normal and pathological, have been discussed more fully than usual. A brief account of the morbid changes in different organs, and their secondary effects, both immediate and remote, has been given. The book can be strongly recommended.

